# Functional diversity shapes the stability of reef fish biomass under global change

**DOI:** 10.1098/rspb.2025.0252

**Published:** 2025-05-14

**Authors:** Lucie Mahaut, Nicolas Loiseau, Sébastien Villéger, Arnaud Auber, Cyril Hautecoeur, Anthony Maire, Camille Mellin, Nicolas Mouquet, Rick Stuart-Smith, Cyrille Violle, David Mouillot

**Affiliations:** ^1^ CEFE, Univ Montpellier, CNRS, EPHE, IRD, INRAE, Montpellier 34090, France; ^2^ CESAB, FRB, Montpellier 34090, France; ^3^ MARBEC, Univ Montpellier, CNRS, Ifremer, IRD, Montpellier 34090, France; ^4^ IFREMER, Unité Halieutique Manche Mer du Nord, Laboratoire Ressources Halieutiques, Boulogne-sur- Mer, France, Boulogne-sur- Mer 62200, France; ^5^ EDF R&D LNHE - Laboratoire National d'Hydraulique et Environnement, 6 quai Watier, Chatou 78401, France; ^6^ Environment Institute, University of Adelaide, Adelaide, South Australia 5005, Australia; ^7^ Institute for Marine and Antarctic Studies, Marine Biodiversity Research Group, University of Tasmania, Hobart, Tasmania 7001, Australia; ^8^ CEFE, Univ Montpellier, CNRS, EPHE, IRD, Montpellier 34090, France; ^9^ Institut Universitaire de France, Paris, France, Paris 75005, France

**Keywords:** community stability, functional traits, ocean warming, fishing

## Abstract

Understanding how environmental and human pressures impact the temporal stability of fish community biomass on shallow reefs is essential for effective conservation and management. These pressures influence community stability directly, by affecting species’ stability and asynchrony in species’ fluctuations. However, their effects may also indirectly depend on the functional traits of the species composing the community, which remains poorly understood. Here, we examine both direct and indirect, trait-mediated effects of environmental variability and human impacts on species’ biomass stability and asynchrony in 215 Australian shallow reefs. These communities span a 10-degree sea surface temperature (SST) gradient and have been monitored over 14 years. Our results indicate higher asynchrony in tropical reefs owing to higher trait diversity and trait redundancy and higher species’ stability in colder, temperate communities owing to higher mean trophic level. Human impacts, through their negative effects on species’ stability and trait diversity, were the main destabilizing factor of fish community biomass. Temporal change in SST destabilized species’ biomass while increasing mean trophic level in fish communities. Overall, our findings show that a comprehensive analysis of the multiple facets of functional diversity is crucial to better understand and forecast the long-term stability of marine ecosystems under global change.

## 1. Introduction

The long-term stability of fish community biomass is a rare shared goal for both biodiversity conservation and sustainable fisheries [1]. The issue is of particular importance in shallow-reef ecosystems whose exceptionally rich biodiversity [2] and productivity [3] sustain food security, economic livelihoods and cultural services [4,5]. In these ecosystems, temporal variations in environmental conditions (e.g. heat waves) and human activities (e.g. fishing) are key drivers of fish community biomass stability [6]. These pressures can indeed increase the demographic variability of fish populations [7,8] and synchronize the temporal dynamics of fish species [9], directly altering the stability of fish community biomass [6]. However, not all species in a community are similarly affected by environmental fluctuations and human activities [10]. The impacts of these pressures depend on the biological characteristics—also known as functional traits—of fishes. For example, larger species from higher trophic levels are more prone to overexploitation while smaller species are more sensitive to changes in sea temperature [11]. These selective, trait-mediated impacts of abiotic stressors can cascade through fish community dynamics by modifying the network of interactions between species [12], with subsequent effects on fish biomass production [13].Therefore, while environmental and human pressures can impact the stability of fish community biomass directly, they can also exert indirect effects by modifying their (functional) composition [14,15]. However, the relative importance of the direct and indirect, biodiversity-mediated effects of environmental and human pressures on the long-term stability of fish community biomass remains to be quantified.

The stability of community biomass is usually measured in the biodiversity–ecosystem functioning literature as the mean annual community biomass divided by its standard deviation, so it is inverse to the coefficient of variation in annual community biomass [16]. Importantly, this index of community stability is the product of two components, namely the average stability of individual species and the asynchrony in species’ biomass fluctuations [17]. The fluctuations of species’ abundances with higher biomass make a larger contribution to the overall community stability than those of more subordinate species. Therefore, the average stability among species weighted by their relative biomass is a key property of community stability [17]. Asynchrony, which depicts negative covariations among species’ biomass fluctuations (also called compensatory dynamics), can stabilize communities through time if the loss in biomass of some species is compensated by gain in biomass of other species [18]. Although increasingly studied in plant communities (e.g. [19,20]), the contributions of average species’ stability and asynchrony, as well as their biotic and abiotic drivers, are largely overlooked in shallow-reef fish communities (but see [6]).

Historically, research into biodiversity–stability relationships has largely focused on the role of species’ richness in regulating the stability of ecosystem functioning (e.g. [16,21,22]). Because the number of species in a community is mathematically linked to the coefficient of variation of total community biomass, species’ richness can stabilize community biomass through a pure mathematical effect (i.e. ‘statistical averaging effect’). The stabilizing effect of species’ richness can also arise from different ecological mechanisms, including biotic interactions, which enhance asynchrony in species’ fluctuations [17]. However, the richness–stability relationship has no universal shape or direction, with empirical studies reporting positive, neutral and negative relationships, notably in reef fish communities [13,23]. This is because species’ richness provides little insight into the different ecological processes involved [17]. By contrast, functional traits capture both species’ responses to environmental variability and species’ roles in community dynamics [24]. As such, trait-based approaches hold great promise to decipher the various ecological mechanisms that underlie the effects of biodiversity on ecosystem functioning [25].

Four facets of the community-level distribution of trait values can mitigate or amplify the effects of environmental and human pressures on ecological stability. The first one corresponds to the dominant trait values within a community, which can be quantified using *Community Weighted Means* (hereafter CWM; table 1). Indeed, if temporal fluctuations in species’ biomass are driven by some traits, the CWM of those traits should affect the average species’ stability [25]. Second, the variability of trait values within a community—or *trait diversity* (table 1)—can promote asynchrony if species with different trait values display different responses to environmental fluctuations [25]. Third, *trait redundancy* (table 1)—which captures the presence of species with similar trait values and is thus likely to fulfil similar functions [36,37]—can enhance community stability by providing an insurance against the loss of individual species [14,38]. Yet, this insurance effect of biodiversity can only operate if redundant species display asynchronous fluctuations through time [25]. Communities composed of species with high redundancy across multiple traits may therefore be less vulnerable to multiple threats [39]. Finally, evidence suggests that certain species might play key roles in community dynamics and ecosystem functioning that cannot be captured by community-aggregated properties [40]. *Trait distinctiveness*, which quantifies the uniqueness of species’ ecological traits [41], can represent such species-specific effects (table 1) [42–44]. Although extreme traits may theoretically promote unique responses to disturbances [34], empirical evidence that functionally distinct species influence the long-term stability of community biomass is lacking.

**Table 1.** Expected relationships between four facets of functional diversity and components of community stability. Community stability is additively partitioned into the average stability of individual species’ biomass and asynchrony in species’ biomass fluctuations.

functional diversity facets	components of community stability	hypothesis	references
community-weighted mean trophic level (CWM_trophic leve_l)	average species’ stability	(a) impacts of climate change are amplified at higher food web levels (b) species from higher trophic levels are more stable	[26] [27]
	asynchrony	predation enhances asynchronous dynamics among prey species	[28]
community-weighted mean maximum length (CWM_length_)	average species’ stability	larger fishes are less sensitive to climate change	[29,30]
community-weighted mean growth rate (CWM_Rate_)	average species’ stability	fast-growing species are less sensitive to disturbances	[31]
community-weighted mean maximum depth (CWM_depth_)	average species’ stability	demersal and reef-associated species have less capacity to persist under disturbances	[32]
trait diversity	asynchrony	species with different traits display different responses to environmental fluctuations, resulting in asynchronous dynamics between species’ biomass	[9,25]
trait redundancy	asynchrony	stabilize community if populations of functionally redundant species have asynchronous fluctuations	[25,33]
number of functionally distinct species (*N* distinct)	asynchrony	functionally distinct species display unique responses to the environment, thus enhancing community-wide asynchrony	[34,35]

Here, we quantified the direct and indirect effects of environmental and human pressures on the temporal stability of fish community biomass. We used an extensive shallow reef monitoring dataset that spans the entire Australian continent during a sea warming period punctuated by multiple extreme marine heatwaves (2008−2021) with observable impacts on fish populations [45]. We focused on 215 fish communities on shallow rocky and coral reefs distributed across Australia, along a latitudinal gradient of mean sea surface temperature (hereafter mean SST gradient) that ranges from 13.9 to 25.4°C (mean = 19.3 **±** 3.1°C) and discriminated species-rich, tropical communities from temperate ones (electronic supplementary material, Figure S1). For each community, we characterized three aspects of environmental variability that capture (i) the linear trend in sea surface temperature through time (hereafter SST change); (ii) the temporal variability in SST caused by increasingly frequent marine heatwaves (hereafter CV_SST_) and (iii) the temporal variability in marine primary productivity, measured by chlorophyll a content (hereafter CV_Chlorophyll_), as it can directly influence the stability of fish communities [46]. We assessed the intensity of human pressures using the human gravity index, which integrates both reef accessibility and human population density, providing a relevant proxy of biomass depletion by fisheries on shallow reef ecosystems [47,48]. We estimated four facets of functional diversity (i.e. CWMs, trait diversity, trait redundancy and trait distinctiveness) using four fish functional traits; species’ maximum length, growth coefficient, trophic level and maximum depth, which relate to both fish species’ responses to abiotic conditions (e.g. deepening) and fish species’ contributions to ecosystem functioning (e.g. food web) [44]. We first analysed the variation of fish community stability, average species’ stability and asynchrony along the SST gradient. Then, we used structural equation modelling (SEM) [49] to quantify the direct and indirect effects of environmental and human pressures on fish community biomass along the SST gradient.

## 2. Material and methods

### (a) Fish distribution data

We use a subset of the dataset published by Edgar *et al*. [45] that comprises abundance data of reef fish species obtained through two long-term reef-monitoring initiatives: Reef Life Survey (RLS; 2007−2021) and Australian Temperate Reef Collaboration surveys (ATRC; 1992−2021). We select sites that have been monitored for at least 7 years during the 2008−2021 period (see details below). In both RLS and ATRC, the size and abundance of all fish species are recorded along 50 m-long transect lines. Multiple transects are surveyed at each site, usually (>95%) between 3 m and 10 m depth (range 0.1 m below low water mark to 42 m). Divers search on both sides of the transect line within a 5 m-wide block recording all fishes, and additionally record cryptic fishes in a close-up search of a 1 m-wide block. Each side of a transect line corresponds to a block. Size classes are defined and abundance (i.e. number of individuals) per size class is visually counted for each species in each block. All surveys were undertaken on shallow reef habitat. The reef habitat varies from kelp-dominated temperate rocky reefs (e.g. Tasmania) to coral-dominated substrate (e.g. Ningaloo Reef). Thus, results can be considered to apply generally to fish communities typical of a range of shallow reef habitats.

### (b) Biomass of fish communities

We define a fish community as all the individuals observed within a site. In order to compute the total annual biomass of each fish community, we first sum the abundances between the two blocks of each transect for each species and size class. Second, we use species-specific length–weight relationships obtained from FishBase [50] to compute the annual biomass of each species in each transect [51]. Then, we calculate the average biomass of each species across transects to have annual species’ biomass at the annual biomass of each species in each community. The number of transect per site varies between 1 and 18. We sum species’ annual biomass to characterize the total, annual biomass of fish communities. In doing so, we have to deal with the fact that field surveys included numerous zero records. Indeed, if a species has been previously observed in a given site, zero records rarely indicate that the species is absent but it rather suggests that the species is present but below survey detection limits. Following Edgar *et al*. [45], if a particular species has been observed in a given site but is absent from at least 1 year of survey, we replace zero counts by the lowest abundance value for that species (i.e. detection limit) at the site across all years divided by 2 (see electronic supplementary material, Figure S5 for sensitivity analysis).

Furthermore, since we aim to analyse the temporal stability of fish community biomass, we confront the challenge that not all sites underwent monitoring throughout the 14 year period. As a result, following the approach outlined by Edgar *et al*. [45], we estimate values for years without survey data by employing a linear interpolation method. This involved assuming that the biomass of a species at a particular site changes in a linear manner during the years that are not surveyed. For times before the initial survey at a site or after the last survey, we assume that the biomass remains constant and is equivalent to the biomass recorded in the nearest surveyed year. Because such interpolation of fish data might strongly affect the temporal dynamics of fish populations—and thus the stability of community biomass—we select sites that have been monitored for at least 7 years during the 2008−2021 period. This number of years is chosen to maximize the number of sites used, while providing the maximum amount of observed annual data for calculating fish biomass and community dynamics. In doing so, we retained 215 sites spanned across Australia (electronic supplementary material, Figure S1) hosting 655 fish species. The mean number of surveyed years per site is 9.05 (electronic supplementary material, Figure S2). We further test the effects of fish biomass interpolation on the stability metrics (details in electronic supplementary material, Figure S6).

### (c) Community stability and its components

For each community of *n* species, we compute the stability of community biomass (*S_c_*) as the inverse of the coefficient of variation of the annual community biomass ${CV}_{c}$:


$$Sc=\;\frac{m_{n}^{c}}{\sqrt{v_{n}^{c}}},$$

with $m_{n}^{c}\;\;and\;\;\sqrt{v_{n}^{c}}$ being the the mean and the standard deviation of the annual biomass of each community *c*, respectively.

We further quantify the average species’ stability weighted by species’ relative biomass [17] as follows:


$$\frac{1}{CV_{sp}}=\;\sum_{i}^{n}\frac{m_{n}^{s}\left(i\right)}{m_{n}^{c}}\;x\;\frac{m_{n}^{s}\left(i\right)}{\sqrt{v_{n}^{s}\left(i,i\right)}},$$

with $m_{n}^{s}(i)$ being the mean of the annual biomass of species *i* in community *c*, and $\sqrt{v_{n}^{s}(i,i)}$ its standard deviation across years.

Finally, we quantify community-wide synchrony, which captures potential compensatory dynamics between the fluctuations of species’ populations, according to Loreau & De Mazancourt [18]:


$$\varphi =\frac{\sum_{i,j}^{S}v_{n}^{s}(i,j)}{\left(\sum_{i}^{S}\sqrt{v_{n}^{s}}(i,i{)}^{2}\right)}$$

The numerator corresponds to the sum of all elements of the variance–covariance matrix of species’ annual biomass, while the denominator is the variance of a hypothetical community with the same species-level variances, but in the presence of perfect synchrony. This index varies between 0 and 1. We use its inverse (i.e. asynchrony; 1 – *φ*), so that 1 indicates perfect asynchrony between the fluctuations of species’ populations. We select this index based on the variance–covariance matrix rather than an index based on the correlations between species’ fluctuations, since a correlative approach is not recommended for relatively short time series (here 14 years; [52]).

Finally, we linearized the mathematical relationship that links the stability of community biomass to average species’ stability and asynchrony following Danet *et al*. [53]:


$$logS_{c}=-logCV_{sp}–1/2log\phi$$

We use the R package codyn [54] to compute the stability of community biomass and asynchrony.

### (d) Functional traits

We describe each fish species using four functional traits, namely maximum length, growth rate, trophic level and maximum depth, based on their expected links with the stability of fish communities (table 1). Maximum length depicts fish body size. Growth coefficient (*year^−^*^1^) expresses the rate at which the asymptotic length is approached [50]. It discriminates the fast-growing species from the slow-growing ones and is linked to metabolism and other life-history traits (e.g. age at maturity). Trophic level summarizes fish diet, with low values indicating preference for feeding on detritus and primary producers and highest values indicating piscivory. Maximum depth identifies species that occupy a greater range in depths. All species in the dataset are found on shallow reefs (1–30 m deep), but species with higher values can live deeper than others, indicating potentially greater access to deep and colder refugia during marine heatwaves. The first three quantitative traits are extracted from FishBase [50], while maximum depth is extracted from [55].

### (e) Facets of functional diversity

First, for the four traits and each community, we compute the mean trait values weighted by species’ relative biomass to test for the dominance effect (i.e. CWM; table 1). We use the average relative biomass of each species within a community over the 14 years of study to weight mean trait values.

Second, we compute the functional distance between species (*d_ij_*) considering the four traits (i.e. maximum length, growth rate, maximum depth, trophic level) simultaneously. Since all these traits are continuous, we use Euclidean distance to compute functional distance between species. Then, we compute trait diversity (FD) and trait redundancy (FR) weighted by species’ relative biomass using the extended framework of functional Hill’s number of order *q =* 1 [56,57].

Hill’s numbers were first developed to assess taxonomic diversity (TD_*q*_) as a number of effective species while controlling the amount of weight given to rare and common species with parameter *q* [58]. With *q* = 1, taxonomic diversity is:

TD_1_ = $expexp\left(-\sum_{i=1}^{S}p_{i}ln(p_{i})\right)$, with *S* being species’ richness and *p_i_* the relative biomass of species *i* in the assemblage.

Chao *et al*. [56] extended this framework for measuring trait diversity based on the distance between species in a functional space. Accordingly, trait diversity relies on parameters *q* and *tau*, the latter defining the threshold level applied to functional distances between species to identify functionally indistinct sets of species. We used *tau* = mean trait distance to optimize scaling of distance between species to compute trait diversity [56]. Trait diversity of order 1 (FD_1_) gives the effective number of equally distinct species and is computed as follows:

.$${FD}_{1}=exp\left(-\sum_{i=1}^{S}p_{i}loglog\left(\sum_{j=1}^{S}\left[1-f\left(d_{ij}(\tau )\right)\right]p_{j}\right)\right)$$

Trait redundancy is expressed as follows [57]:


$$FR_{1}=1-FD_{1}/TD_{1}.$$

FR_1_ indicates that the observed community is as redundant as a community of equally abundant species where a proportion equal to *R*_1_ could be removed without impacting the effective trait diversity of order *q* = 1.

We use the alpha.fd.hill function of the mFD R library [59] to compute trait diversity and trait redundancy.

Finally, we quantify trait distinctiveness—that is, the originality of a species’ combination of traits compared with other species in an assemblage [41]—as followed:


$$D_{i}=\;\sum_{j=1,\;j\neq i}^{S}d_{i,j}.$$

with *d_i,j_* being the Euclidean distance of each pairwise species in the functional space made of species’ length, trophic level and maximum depth. Trait distinctiveness of a given species can be computed either within the whole species’ pool (i.e. global distinctiveness) or within each community (i.e. local distinctiveness). We find a strong correlation between these two values (*r* = 0.9, electronic supplementary material, Figure S9) and thus consider only global trait distinctiveness in the following. Accordingly, we define functionally distinct species as the 25% of the 655 species that have the higher global trait distinctiveness. Then, we compute the number of functionally distinct species that occur within each community (*N* distinct). Because the number of functionally distinct species may be directly proportional to species’ richness, we compute linear regression between *N* distinct and species’ richness and consider the residuals of this regression to quantify the number of functionally distinct species [60]. We used the R library funrar [61] to compute trait distinctiveness.

We log-transform the length at maturity and maximum depth before the computation of trait diversity, trait redundancy and trait distinctiveness.

### (f) Environmental factors and human impacts

Annual sea surface temperature (SST) and annual chlorophyll a content were retrieved from the Coral Reef Watch Satellite monitoring program (see electronic supplementary material, Table S3). The data are a 5 × 5 km raster for each year between 2008 and 2021.

We compute the temporal mean of annual SST for each community to characterize the gradient of sea temperature. In addition, we compute two indicators of climate change. First, we quantify temporal changes in SST using linear models with ‘annual SST’ and ‘year’ as dependent and independent variables, respectively. We use the coefficient of regression of ‘year’ to quantify the annual rate of change in SST. Second, we assess the variability of change in SST by computing the coefficient of variation of SST across years (CV_SST_). In addition, we account for temporal change in chlorophyll a content since change in ocean primary productivity can directly affect the biomass of fish communities through bottom-up processes [46]. We characterize year-to-year variability in chlorophyll a content using the coefficient of variation of annual chlorophyll a content. We also compute mean annual chlorophyll a and linear change in chlorophyll a but as these variables were strongly correlated with SST (*r* > 0.7, data not shown) they were not kept in further analyses. Finally, for each community, we compute site depth by averaging the depth of all the transects present in a given site.

We use the human gravity index [47] to quantify the intensity of human impacts on marine ecosystems. This index is calculated as the ratio between human population density divided by travel time. Gravity measures the intensity of human impacts in the surrounding seascape. It has been shown that gravity diminishes the effectiveness of marine reserves at sustaining reef fish biomass and the presence of top predators, even where compliance with reserve rules is high [47]. It is also strongly related to biomass production and turnover in fish communities [3], and more importantly, to the fisheries’ impact on fish biomass [48,62].

### (g) Statistical analyses

We first tested for differences in community stability, average species’ stability and asynchrony along the mean SST gradient using linear regressions. We found that mean SST represented a biogeographic gradient that distinguished species-rich northernmost tropical communities from southern species-poor and temperate communities (electronic supplementary material, Figure S1). Community and average species’ stability were log-transformed for normality assumption.

Then, we use structural equation models (SEM) to quantify the relationships between abiotic factors (i.e. human impacts, mean SST, SST change, CV_SST_, CV_chlorophyll_, *N*_years_ and site depth), facets of functional diversity (i.e. CWMs, trait diversity, trait redundancy and number of functionally distinct species) and temporal stability of community biomass. SEMs represent a powerful way to disentangle complex mechanisms controlling biodiversity–stability relationships, including direct and indirect effects of the environment (e.g. [6,53]). Community stability is the sum of average species’ stability and asynchrony. Thus, in the SEM, the response variable ‘community stability’ has only two explanatory variables, which are its two components. We accounted for the correlation between average species’ stability and asynchrony in the SEM [17]. Then, we assume that abiotic factors and functional diversity facets can directly affect asynchrony and average species’ stability. The expected relationships between the different facets of functional diversity and the two components of community stability are theoretically grounded and are summarized in table 1. In addition, we assumed that each facet of functional diversity can be correlated to the abiotic factors. Finally, we accounted for pairwise correlations between the different facets of functional diversity in the SEM (all correlations are < 0.6; electronic supplementary material, Figure S3).

Because there is no automatic process to select significant variables in SEM, first we run linear models to identify the most important covariates for each response variable (except community stability) included in the SEM (i.e. average species’ stability, asynchrony and the four facets of functional diversity; outputs in electronic supplementary material, Figure S4 for all facets of functional diversity). Indeed, preliminary analyses have shown that a potentially important explanatory variable can be considered as non-significant from the SEM outputs when integrating all explanatory variables in all regressions of the SEM. This was owing to the existence of correlations between explanatory variables (although never greater than 0.6; electronic supplementary material, Figure S3). We use the *dredge* function from the MuMIn R library [63] to select significant variables. Then, for each of the linear regressions of the SEM, we only consider covariates that have been previously selected. In doing so, we limit the number of covariates implemented in the SEM and avoid misinterpretation of the results that would otherwise only rely on the *p*-values calculated with the SEM. CWM_growth_ coefficient and trait distinctiveness were not included in the SEM because they had no significant effect on any stability component, so they cannot mediate the effects of abiotic factors on community stability. We assess the goodness of fit of the SEM using the log-likelihood test implemented in the piecewise SEM R library [49]. This test allows us to detect whether a significant path is missing from the SEM.

## 3. Results

Linear regressions indicated that average species’ stability and asynchrony significantly varied along the biogeographical gradient captured by mean SST. More precisely, average species’ stability decreased (estimate = −0.02; *p* = 0.01), while asynchrony increased (estimate = 0.02, *p* = 0.01), with increasing mean SST. Community biomass stability did not show significant variation along the mean SST gradient (estimate = −0.001; *p* = 0.92).

**Figure 1.**
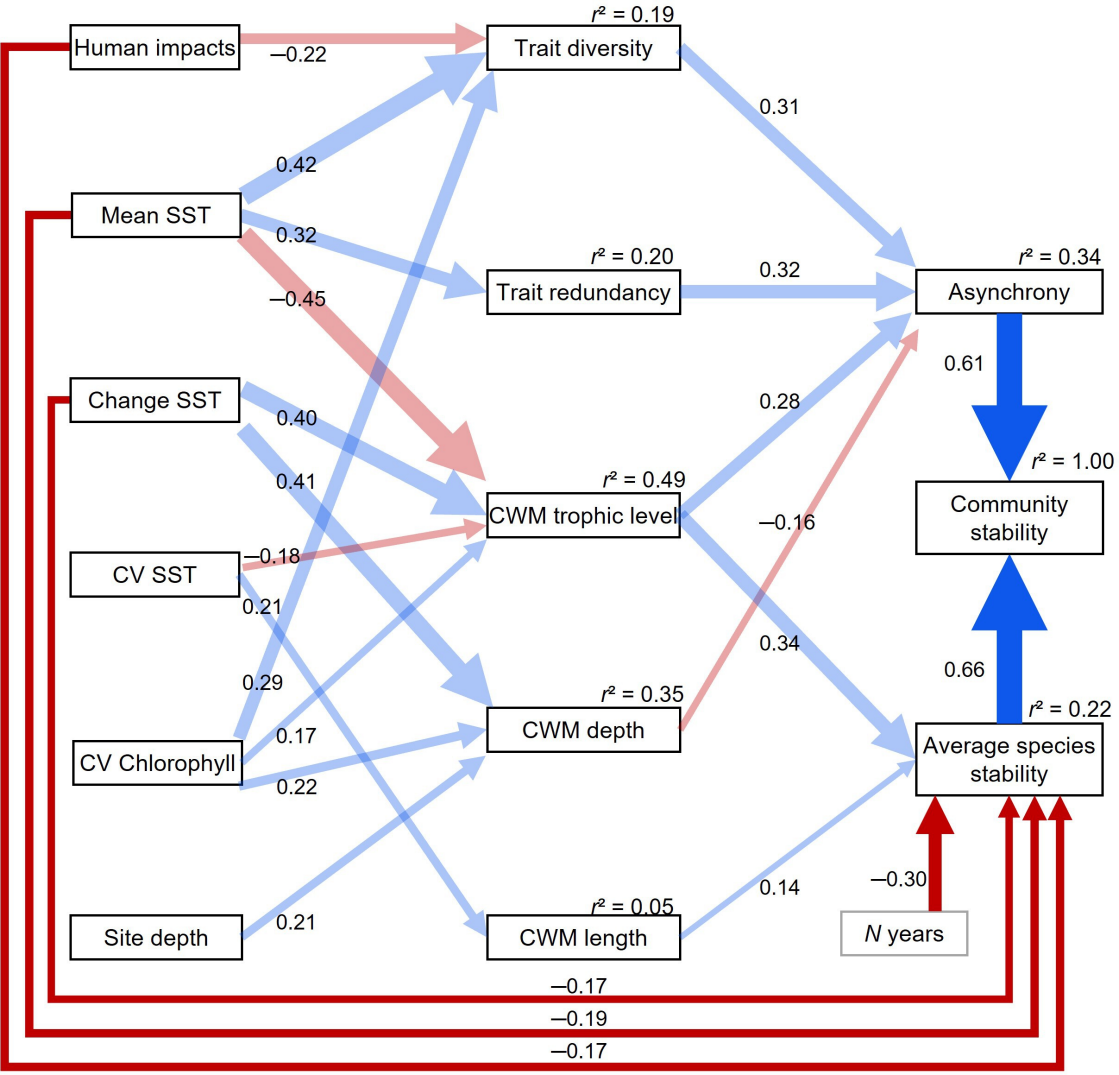
Structural equation model relating abiotic factors, functional diversities and temporal biomass stability of shallow reefs fish communities. Abiotic factors include mean annual sea surface temperature (SST), site depth, SST change, CV_SST_ and CV_Chlorophyll_ as well as human impacts. Functional diversity facets include trait diversity, trait redundancy, Community Weighted Mean (CWM) values of trophic level, maximal depth and maximum length. Only the variables exerting significant effects and significant relationships are shown. The sizes of arrows are proportional to the effects, with path coefficients indicated. Blue lines: positive relationships. Red lines: negative relationships. Dark colours: direct relationships. Light colours: indirect effects. *r*² indicates the proportion of the variance explained by the structural equation model in the response variables. It equals one for community stability since it is a deterministic relationship with asynchrony and average species’ stability. The goodness of fit is evaluated through the Fisher’s *C* statistic (*p* = 0.41), indicating that the hypothesized model structure is supported by the data and that no significant path is missing.

Results from the SEM showed that average species’ stability and asynchrony were equally important stabilizing mechanisms for fish community biomass (figure 1). However, the two components of community stability displayed different responses to environmental and human pressures.

Average species’ stability was directly reduced by human impact and SST change (figure 1). In addition, abiotic factors exerted indirect effects on average species’ stability by modulating the community weighted mean trait values (figure 1). More precisely, average species’ stability increased with the community mean trophic level (CWM_trophic level_) and maximum length (CWM_length_). CWM_trophic level_ increased with SST change and CV_Chlorophyll._ .Therefore, SST change and CV_Chlorophyll_ exerted positive indirect effects on average species’ stability. In addition, CV_SST_ promoted CWM_length_ but reduced CWM_trophic level_ and as such influenced average species’ stability both positively and negatively.

Opposite to average species’ stability, asynchrony in species’ biomass fluctuations only experienced indirect effects of abiotic factors, which operated through different facets of functional diversity (figure 1). Asynchrony increased with trait diversity, trait redundancy and mean trophic level but decreased with mean species’ maximum depth (CWM_depth_). Human impacts and CV_SST_ reduced asynchrony by lowering trait diversity and CWM_trophic level_, respectively. SST change enhanced species’ asynchrony by increasing CWM_trophic level_ (figure 1). However, it also promoted the dominance of depth generalist species that were also found on deeper reefs (higher CWM_depth_), and thereby negatively affected asynchrony. Similarly, CV_chlorophyll_ had both positive and negative effects on asynchrony, which were mediated by its positive effects on trait diversity and on CWM_trophic level_ and CWM_depth_. Community trait distinctiveness and mean species’ growth coefficient (CWM_growth_) were the only functional diversity facets that had no significant effect, on either average species’ stability or asynchrony.

Our findings further revealed that different mechanisms could stabilize fish communities along the SST gradient (figure 1). First, average species’ stability decreased with mean SST. This direct effect of sea surface temperature on species’ stability was amplified by the negative influence of mean SST on CWM_trophic level_. Conversely, trait redundancy and trait diversity both increased with mean SST. Higher trait diversity and redundancy in warmer seas can therefore promote asynchrony, while in cooler waters, compensatory dynamics mostly arise from the dominance of species with higher trophic level.

**Figure 2.**
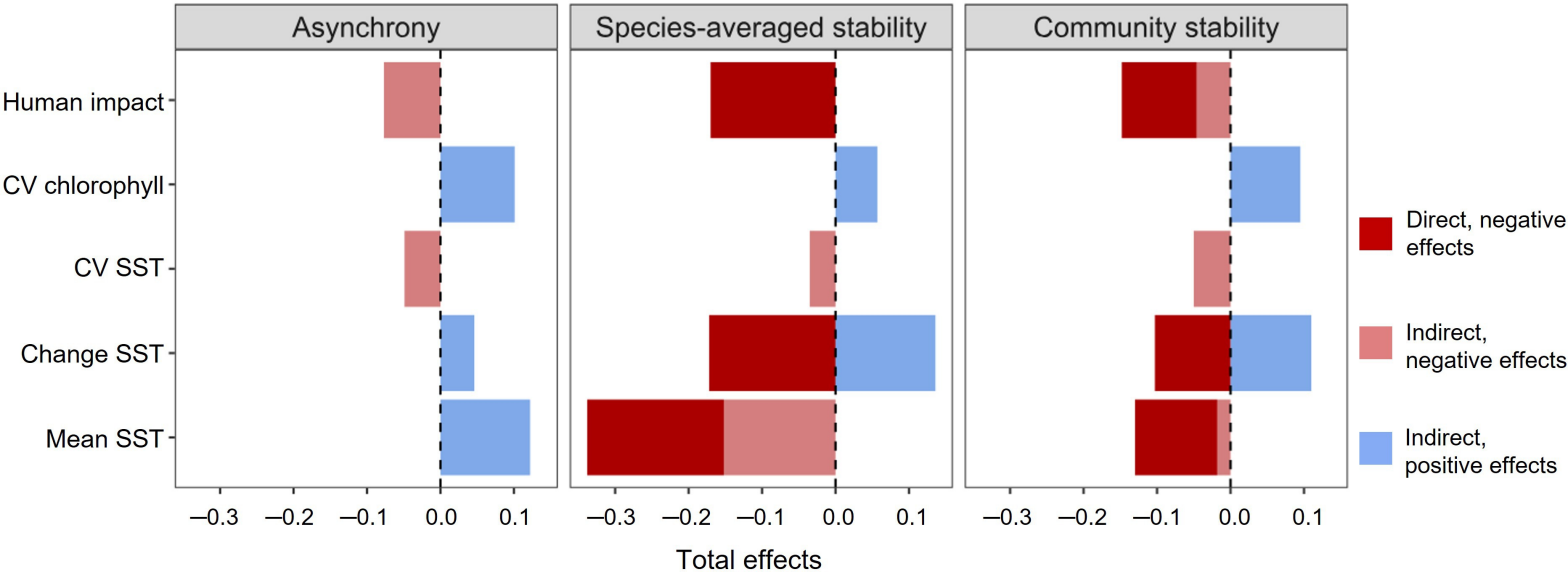
Total accumulated effects of abiotic factors on fish biomass asynchrony, average species’ stability and community stability. Total indirect effects on asynchrony and average species’ stability are computed by multiplying and summing the path’s segments that involve one or more abiotic factor in the structural equation model shown in figure 1. Total effects on community stability correspond to the weighted sum of the effects on asynchrony and average species’ stability. Dark and light red: direct and indirect negative effects of abiotic factors, respectively. Light blue: indirect positive effects of abiotic factors.

To synthesize the findings of the SEM, we quantified the total direct and indirect effects of abiotic factors on average species’ stability, asynchrony and community stability (figure 2). These results highlighted that the intensity of human impacts and mean sea temperature were the main destabilizing factors of fish community biomass (figure 2). They also indicated that positive trait-mediated effects of SST change on both average species’ stability and asynchrony can compensate for its direct destabilizing effect on fish community (figure 2). By contrast, the overall effects of CV_SST_ on the different facets of functional diversity contributed to destabilizing fish community biomass (figure 2).

Finally, these results were robust to several uncertainties and potential biases. First, as some fish communities were not monitored annually between 2008 and 2021, we interpolated the biomass of fish species for the missing years (details in §2). We carried out a sensitivity analysis to assess the correlation between community stability quantified using complete time series only (i.e. 14 years, *n* = 25) or degraded versions of these time series where we randomly selected some years and then substituted the raw values with interpolated ones. In the worst-case scenario, for a given site with 7 years of missing data, we found that stability computed from raw data and from gap-filled data correlated at *r* = 0.66 (electronic supplementary material, Figure S6). We accounted for this potential bias in the estimation of community stability in the SEM by controlling for the number of years a community was surveyed (hereafter *N*_years_, mean = 9.0 ± 2.4 years). Results indicated that the greater the amount of gap-filled data, the stronger was the average species’ stability (figure 1). Second, we controlled for site depth in the SEM since the depth at which each community was monitored varied from one site to another (mean = 6.5 ± 2.9 m). Site depth only indirectly affected asynchrony by increasing CWM_depth_ (figure 1). Lastly, we checked for spatial-autocorrelation in the SEM residuals and found no significant signal (electronic supplementary material, Table S2).

## 4. Discussion

This study demonstrates that several facets of functional diversity mediate the effects of environmental and human pressures on the temporal stability of fish community biomass. We show that temporal change in SST and human impacts are key drivers of species’ instability, while functional diversity, through its different facets, largely controls asynchrony between species. Importantly, trait-mediated pathways can either mitigate or amplify the direct destabilizing effects of environmental changes and human impacts on community stability. While the plurality of functional diversity has long been recognized in biodiversity–ecosystem functioning research [64], it remains largely ignored in the biodiversity–stability literature (but see [19]). Our results demonstrate the promise of the multi-faceted trait-based approach to understanding the long-term stability of ecosystem functioning.

Strikingly, we find a considerable direct destabilizing effect of human impacts, approximated by human gravity, on species’ biomass stability, and subsequently on fish community stability. This echoes a recent finding showing a lower stability of reef fish communities most accessible to humans [6] and probably reflects the fact that fishing amplifies the natural fluctuations of fish populations through multiple pathways [7,8]. Furthermore, we show that human impacts exert negative effects on trait diversity, in line with Benedetti-Cecchi *et al*. [6], who reported higher functional richness in reef fish communities located in marine protected areas. However, contrary to the findings of Benedetti-Cecchi *et al*. [6], our results indicate that trait diversity is a main driver of species’ asynchrony, as expected under the niche complementarity hypothesis [25]. Such a discrepancy between the two studies certainly arises from differences in the way that trait diversity was characterized. Trait richness, as used in [6], does not take into account potential differences in the relative abundance of species, whereas trait diversity, as used in this study, does. Therefore, our results suggest that complementarity mechanisms (i.e. asynchrony) between abundant species stabilize community biomass, highlighting the crucial role played by species’ evenness [17]. These findings are also key for understanding the long-term effects of human impacts in general—and fishing in particular—on compensatory dynamics in reef fish communities. Previous studies show that fishing might enhance asynchronous fluctuations between prey and predator species [65], or conversely, synchronize fish population responses to environmental drivers [66]. Our results suggest that such a synchronization between fish biomass fluctuations might be caused by the reduction of the species’ trait diversity that is needed to support asynchronous dynamics. Yet, a more balanced harvest between fish functional groups can be effected by diversifying fishing gears or practices to maintain asynchrony within reef fish communities [67]. Finally, the decline in the range of trait values under disturbance can be linked to the human-induced erosion of functionally distinct species and certain evolutionary lineages [68,69]. Accordingly, we find that the number of functionally distinct species strongly decreases with rising human impact (electronic supplementary material, Figure S4), suggesting that functional distinctiveness can be used as an indicator of anthropogenic pressures on communities.

Investigating the roles of different facets of functional diversity along a wide biogeographic gradient shows that different mechanisms can generate compensatory dynamics among reef fishes. More specifically, asynchrony increases with trait diversity and redundancy, which are both higher in warmer seas [14]. The positive relationship between trait redundancy and asynchrony echoes the ‘insurance hypothesis of biodiversity’, which states that trait redundancy buffers the functioning of ecosystems against the loss of a few species, as long as redundant species display asynchronous fluctuations [38]. Competition between functionally redundant species can also generate compensatory dynamics under fluctuating environments. In that perspective, competition between species sharing the same traits can reduce the abundance of the lower competitor species, which in turn can facilitate the increase of another species, as previously observed [70]. In addition, we find that the mean trophic level of fish communities in colder seas is higher, on average. Accordingly, recent findings in Australian reef ecosystems indicate that the proportions of invertivores, and to a lesser extent of piscivores, are higher in fish communities of colder seas [71]. Given that fish communities characterized by high mean trophic level display higher asynchrony, these results might indicate that predation can generate compensatory dynamics between the fluctuations of prey species in cold waters [28]. Meanwhile, lower mean trophic level in warmer seas results from the dominance of herbivores and planktivores [71], which are known to migrate towards more southerly locations to track ocean warming (i.e. ‘tropicalization’ of temperate fish communities, [72]). Accordingly, we observe higher diversity of species’ biogeographic origins (i.e. tropical, warm and cool; [45]) in southern fish communities (electronic supplementary material, Figure S7). The mix of warmer- and cooler-affinity species in temperate fish communities can therefore also generate asynchrony in these communities. Accounting for untested factors such as species’ thermal affinity or migratory capacity will allow testing of this hypothesis. Finally, the structural complexity of tropical coral reefs may differ from that of more temperate, rocky reefs, with critical effects on fish community stability. Indeed, habitat complexity determines the distribution of fish size [73], the abundance of piscivorous and mobile planktivory species [74], predator–prey interactions [58] and the structure of food webs [75] in reef ecosystems. By modulating these different facets of fish biodiversity, habitat complexity can thus contribute to the temporal stability of fish biomass [76]. However, characterizing habitat complexity is a grand challenge as it is a multidimensional and multiscale concept [77]. Furthermore, different properties can generate habitat complexity in tropical (e.g. coral cover) and temperate (e.g. algae cover) reef ecosystems. The global assessment of the structural complexity of shallow-reef ecosystems will therefore be of crucial importance for evaluating its role in ecosystem functioning and stability. Overall, we report higher community stability in colder water, principally owing to lower variability in species’ biomass. This result shows that across the SST biogeographic gradient, species’ richness, which is far higher in tropical communities than in colder, temperate reefs, has no stabilizing effect on community biomass. Yet, temperate communities might become more unstable in the coming years given that climate change—and more particularly the increase in sea surface temperature—is more pronounced in temperate than in tropical marine ecosystems [78]. Accordingly, we observe that increasing sea temperature through time directly reduces average species’ stability, as previously reported [6]. However, we also find that increasing sea temperature through time increases mean community trophic level, and as such, indirectly enhances average species’ stability and asynchrony. This finding is particularly important given that this trait-mediated effect can compensate for the direct destabilizing effect of SST change. Additional analyses suggest that rising sea temperatures also lead to an increase in the average trophic level because low-trophic-level species experience a greater decline in biomass over time, while high-trophic-level species are less affected by temperature changes (electronic supplementary material, Figure S8). These results also link with the fact that mean community body size increased as the year-to-year variability in SST increased. Our findings thus collectively suggest a warming-induced increase in the average stability of fish species on cooler temperate reefs through increased mean body size and trophic level. In other words, those larger, more generalist species handled local warming best [79]. Yet, increasing mean community trophic level with increasing sea temperature through time is apparently contradictory to the spatial pattern of higher mean trophic level of fish communities in cooler seas. The temporal pattern may be the reverse of the spatial one simply because the magnitude of temporal warming (i.e. mean = 0.1 ± 0.2°C per year) is much smaller than the spatial gradient covered (from 13.9 to 25.4°C) and if temporal change in SST induces a shift in the dominance of local species (electronic supplementary material, Figure S8).

To conclude, our findings emphasize the need to concomitantly address species-level stability, compensatory dynamics and multiple facets of functional diversity to grasp the effects of environmental changes and human impacts on the long-term functioning of marine ecosystems. While the use of trait-based approaches has recently been questioned [80], the overwhelming influences of the different facets of functional diversity, together with the fact that community stability was higher in temperate, species-poor communities, confirm the great potential of traits to improve our understanding of biodiversity–stability–environment relationships across taxonomic groups. Importantly, our results indicate that different mechanisms promote fish biomass stability in temperate and tropical reefs. Integrating this knowledge into ecosystem management programmes will help to conserve fish biodiversity while improving the sustainability of fisheries.

## Data Availability

Raw data are publicly available in the URLs provided in electronic supplementary material, table S3 [81]. The process data are available at [82]. The code used to analyse these data is at [83]. Any additional information required to reanalyse the data reported in this paper is available from the lead contact upon request.
